# How to Show the Real Microbial Biodiversity? A Comparison of Seven DNA Extraction Methods for Bacterial Population Analyses in Matrices Containing Highly Charged Natural Nanoparticles

**DOI:** 10.3390/microorganisms3040695

**Published:** 2015-10-20

**Authors:** Rene Kaden, Peter Krolla-Sidenstein

**Affiliations:** 1 Department of Medical Sciences, Clinical Microbiology, Uppsala University, Dag Hammarskjöldsväg 17, Uppsala 75185, Sweden; 2 Chemistry of Oxydic and Organic Interfaces, Institute of Functional Interfaces (IFG), Karlsruhe Institute of Technology (KIT), Herrmann-von-Helmholtz-Platz 1, Eggenstein-Leopoldshafen 76131, Germany; E-Mail: peter.krolla-sidenstein@kit.edu

**Keywords:** bacteria, biodiversity, DNA-extraction, ecology

## Abstract

A DNA extraction that comprises the DNA of all available taxa in an ecosystem is an essential step in population analysis, especially for next generation sequencing applications. Many nanoparticles as well as naturally occurring clay minerals contain charged surfaces or edges that capture negatively charged DNA molecules after cell lysis within DNA extraction. Depending on the methodology of DNA extraction, this phenomenon causes a shift in detection of microbial taxa in ecosystems and a possible misinterpretation of microbial interactions. With the aim to describe microbial interactions and the bio-geo-chemical reactions during a clay alteration experiment, several methods for the detection of a high number of microbial taxa were examined in this study. Altogether, 13 different methods of commercially available DNA extraction kits provided by seven companies as well as the classical phenol-chloroform DNA extraction were compared. The amount and the quality of nucleic acid extracts were determined and compared to the amplifiable amount of DNA. The 16S rRNA gene fragments of several taxa were separated using denaturing gradient gel electrophoresis (DGGE) to determine the number of different species and sequenced to get the information about what kind of species the microbial population consists of. A total number of 13 species was detected in the system. Up to nine taxa could be detected with commercially available DNA extraction kits while phenol-chloroform extraction lead to three detected species. In this paper, we describe how to combine several DNA extraction methods for the investigation of microbial community structures in clay.

## 1. Introduction

According to the soil type classification, soils consists mainly of sand, silt, and clay that are often mixed. Clay minerals are natural fine-grained phyllosilicates that might occur in soils. Thus, only soils with a high amount of clay minerals or soils with a defined small grain size are defined as clays. Depending on the discipline, nanoparticles with a particle size less than 4 µm (sedimentologists), 2 µm (geologists), or 1 µm (colloid chemists) are defined as clay particles. All clays are generally plastic if they contain water and they harden if they dry or when fired. This principle is applied in the clay industry. The plasticity might even be influenced by microorganisms. Many well-known methods exist to analyze the microbial population structure in soils, but only a few are applicable for industrially used clays. Some of the approaches are based on the cultivation of microorganisms, on methods of molecular biology, or the combination of both [1,2,3]. Only 0.1%–0.5% of all microbial species are detectable with culture-dependent methods [4]. In contrast to these methods, up to 80% of the bacterial fraction could be detected with approaches based on molecular analyses [5,6,7]. These culture-independent methods are highly sensitive and are applicable to prove the biodiversity of a system with the exception of ecosystems in clayey soils, especially with clays exhibiting a highly charged mineral surface. Those charges combined with bi- or multivalent cations inhibit an entire extraction of negatively charged DNA by common methods [8]. This effect may be enhanced in the presence of humic substances or other charged biomolecules [9]. To saturate the particle charges, different methods are already published that are mainly based on the addition of artificial nucleic acid molecules, allochthonous DNA, or skim milk powder [10,11]. The majority of the described methods are validated according to the amount of extracted DNA or the ability to amplify the DNA in polymerase chain reaction (PCR). However, the detection of a high number of taxa is most important for ecological research. Within DNA extraction the cell walls of gram-positive bacteria and Mycobacteria are more resistant than those of gram-negative bacteria. This leads to a shift or lack in detection of special groups of microorganisms.

The aim of this study was the detection of as many taxa as possible with several commercial kits as well as with phenol-chloroform extraction with and without the addition of artificial DNA or skim milk powder to provide a method or a procedure for DNA-based population analysis in clays.

## 2. Experimental Section

### 2.1. Materials

A low-silt, industrially used clay from Westerwald, Germany was provided by Sibelco Germany GmbH and prepared with a final water content of 50%. A sample of 50 g was homogenized and frozen in aliquots 1 g each. All aliquots were analyzed in a parallel approach. The detailed mineralogical characterization of the clay that was applied in this study was published by Petrick *et al.* [12]. 

### 2.2. Methods

With the aim to compare methods that are based on different procedures, DNA extraction kits with and without mechanical treatment as well as commercial kits with different chemicals for cell lysis and DNA cleaning were chosen. A modification of a few methods was tested in a parallel approach to the manufacturer’s instructions (see detailed description below each method). Furthermore, a classical phenol-chloroform extraction was tested. A summary of all methods and variations including the abbreviations used in the text is shown in Table 1.

**Table 1:** Compared methods and variations.

Method	Mechanical Disruption	Company	Abbreviation
Fast DNA Spin Kit for soil	X	MP Biomedicals	MP Soil
Fast DNA Spin Kit for soil modified (EtOH)	X	MP Biomedicals	MP Soil EtOH
innu speed soil DNA Kit Sample 1	X	Analytik Jena	AJ Soil 1
innu speed soil DNA Kit Sample 2	X	Analytik Jena	AJ Soil 2
Precellys Soil DNA Kit	X	PeQLab	PeQLab Soil
QIAamp DNA MiniKit slurry extract	-	Qiagen	QIA Mini slu
QIAamp DNA MiniKit supernatant extract	-	Qiagen	QIA Mini sup
QIAamp DNA stool Kit User developed Sample 1	-	Qiagen	QIA stool 1
QIAamp DNA stool Kit User developed Sample 2	-	Qiagen	QIA stool 2
Soil Microbe DNA Kit Sample 1	X	Zymo Research	ZR Soil 1
Soil Microbe DNA Kit Sample 2	X	Zymo Research	ZR Soil 2
Pure Skim Milk Powder	X		Skim Milk
Phenol-Chloroform extraction without Skim Milk	X		PhChl
Phenol-Chloroform extraction with Skim Milk 1	X		PhChl Skim 1
Phenol-Chloroform extraction with Skim Milk 2	X		PhChl Skim 2
GeneMATRIX Soil DNA Purification Kit + 0 µL Poly A	X	EURx	EURx Soil 0 Pol
GeneMATRIX Soil DNA Purification Kit + 20 µL Poly A	X	EURx	EURx Soil 20 Pol
GeneMATRIX Soil DNA Purification Kit + 40 µL Poly A	X	EURx	EURx Soil 40 Pol
GeneMATRIX Soil DNA Purification Kit + 60 µL Poly A	X	EURx	EURx Soil 60 Pol

X, mechanical cell disruption was applied; -, not applied.

**Phenol-Chloroform Extraction.** The extraction of nucleic acids based on chemical treatment with phenol chloroform was described by Tsai and Olson [13] and was modified for this study. The extraction buffer contained w/v values of 1.48% Tris HCl, 3.27% ethylene diamine tetraacetic acid (EDTA), 8.76% NaCl, and 1.56% NaH_2_PO_4_. A slurry of 1 g sample and 2 mL extraction buffer containing 50 µL lysozyme (100 mg/mL) and 100 µL proteinase K (20 mg/mL) was incubated 60 min at 37 °C on a “Enviro genie” overhead-shaker (Scientific Industries, New, York, NY, USA). To saturate the charges of the matrix particles, 4% w/v skim milk powder was added to the extraction buffer for a second approach. To estimate the possible influence of skim milk powder, a DNA extraction from pure 4% w/v skim milk powder was accomplished in the same way as the clay samples were treated. All samples were centrifuged for 5 min at 2500 rpm and phenol-chloroform-isoamylalcohol (25:24:1) was added to the removed supernatant in a 1:1 ratio. The vials were shaken by hand for 3 min at 20 °C and subsequently centrifuged for 3 min at 1500 g and 20 °C. The upper phase was transferred in a tube with the equal volume of chloroform-isoamylalcohol (24:1). After centrifugation for 3 min at 1500 g and 20 °C, 0.6 vol. isopropanol and 0.1 vol. sodium acetate (3 M, pH 5.2) were added to the transferred aqueous phase. DNA precipitation was done by overnight incubation at 4 °C and a subsequent centrifugation for 1 h at 10,000 g and 4 °C. After discarding the upper phase, the DNA pellet was cleaned with 1 mL ice-cold ethanol (70%). The samples were centrifuged at 10,000 g for 25 min at 4 °C and the supernatant was removed. To dry the pellets, the vials were stored 1 h at room temperature in a laminar airflow box to avoid contaminations. The DNA pellets were dissolved in 50 µL DNA-free PCR water.

Fast DNA Spin Kit for Soil (MP BIOMEDICALS, Santa Ana, CA, USA). The DNA extraction was accomplished according to the supplier’s instructions. The matrix-bound DNA was purified with 98% ethanol in addition to the intended cleaning steps within a second experiment.

QIAamp DNA Mini Kit (Qiagen, New York, NY, USA). Since many microorganisms in clay are associated with charged particles, the DNA from one sample was extracted directly from the slurry and another sample from the supernatant after centrifugation at 10,000 g for 1 min.

Gene MATRIX Soil DNA Purification Kit (EURx, Gdansk, Poland). With the aim to saturate the charges of the clay particles, 60 µL, 40 µL, and 20 µL Poly A (100 pmol/µL) were added per 1 g sample prior to cell lysis in addition to a fourth extraction according to the manufacturer’s protocol.

Innu Speed Soil DNA Kit (Analytik Jena, Jena, Germany, Precellys Soil DNA Kit (PeQLab, Erlangen, Germany), QIAamp DNA Stool Kit (Qiagen), Soil Microbe DNA Kit (Zymo Research, Orange, CA, USA). These kits were applied according to the instructions provided by the supplier.

### 2.3. DNA Analysis

The concentration and quality of the extracted DNA was determined using a Nanodrop ND1000 spectrophotometer (Thermo Fisher Scientific, Waltham, MA, USA). A 16S rRNA-directed qPCR was applied to appraise the influence of inhibitors and the quality of DNA for further molecular analysis. The primer set 27f [14] and 517r [15] were applied in combination with SYBR^®^ Green (Applied Biosystems, Carlsbad, CA, USA) as detection reagent. The PCR program was 10 min at 94 °C, 40 cycles of: 1 min at 94 °C followed by 1 min at 58 °C, 1.5 min at 72 °C, and an endelongation for 10 min at 72 °C. The qPCR results were analyzed using the SDS 1.2.3 software provided by Applied Biosystems. All samples were analyzed on the same plate to allow comparing the results. In addition to an undiluted sample that was obtained directly from DNA extraction, dilutions of 1:10, 1:100 were applied for qPCR for each method.

The qPCR amplificates were directly used for denaturing gradient gel electrophoresis (DGGE) [16]. A vertical gel with a urea gradient of 40%–70% in combination with a run time ratio of 1000 Vh was used to separate the 16S rRNA genes of the amplified samples according to their sequences.

Single bands for sequencing were recovered with a scalpel while the gel was placed on a UV illumination plate. Diffusion of DNA fragments took place by incubation of the gel slides in 25 µL H_2_O for at least 12 h at 8 °C. For amplification of the DNA, a further PCR was applied as described above using the same primers and a similar program with 25 cycles. The samples were subsequently sequenced by GATC biotech AG. The NCBI database and the algorithm blastn were used [17] for taxa determination.

## 3. Results and Discussion

The concentration and quality of all DNA extracts are outlined in Table 2. The iron content in the samples was very low and might have no effect on the absorbance determination of the DNA [12].

The extracted amounts of DNA varied between 0.1 µg/g and 19.6 µg/g sample. The highest DNA recovery rates were observed using the QIAamp DNA stool Kit (Qiagen) and Soil Microbe DNA Kit (Zymo Research), while the smallest amount was obtained using phenol-chloroform extraction. The quality of the extracted DNA was determined by calculation of the ratio λ260/λ280, which results in 1.8 up to 2.0 for clean DNA, and λ260/λ230 with an expected result between 2.0 and 2.2. Coextracted proteins may decrease the ratio λ260/λ280 and phenols as well as carbohydrates have an analog influence on the quotient λ260/λ230. In 18 of 19 samples, a λ260/λ230 ratio below 1 was detected. There might be high amounts of humic substances in the matrices and it was not possible to remove them with the majority of the considered methods. Because of the similarity of DNA and some humic substances, it was almost impossible to separate both types of molecules during DNA extraction. Only in one phenol-chloroform-extracted sample with added skim milk was the expected ratio λ260/λ230 higher than 2. However, this sample contained a high amount of coextracted proteins that led to a quotient λ260/λ280 of 0.89. The poor quality of a few samples resulted in a strong inhibition of the qPCR. A summary of all obtained cycle threshold (Ct) values is shown in Table 2. In many cases, only a dilution of the extracts resulted in amplification of DNA. Through dilution there was a decreased amount of inhibitors, but also less DNA molecules in the sample.

**Figure 1:**
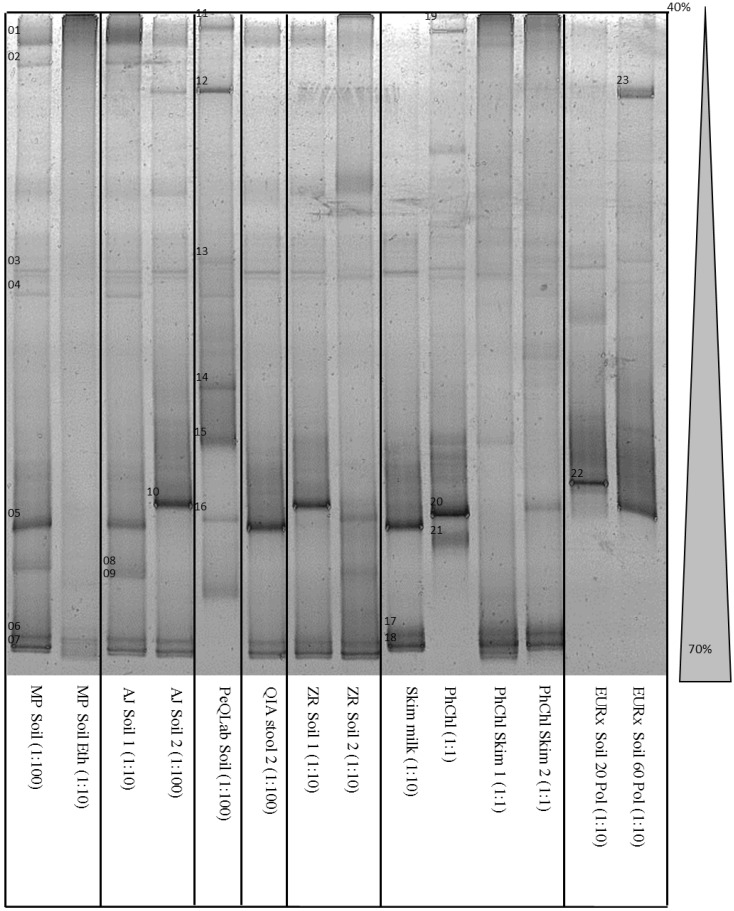
DGGE of 16S rDNA fragments obtained by several DNA extraction methods; DNA dilution for PCR in parentheses.

The aim of the dilution series was to find at least one dilution for each method to obtain a PCR product. There was no method in which all three dilutions led to a PCR product. At least one PCR product from one dilution was amplified from 14 of 19 samples or variations. This leads to the recommendation to apply several dilutions of DNA extract for further PCR analysis for investigation of challenging matrices. DNA from the samples extracted with the phenol-chloroform method could be amplified from several dilution levels but also with a low cycle threshold (Ct) compared to the other methods. All samples with no PCR product were analyzed again, yielding the same results. The samples with the lowest Ct value obtained by one method or variation were used for a subsequent DGGE (Figure 1).

**Table 2:** Quality and amount of extracted DNA and Ct-values of qPCR.

Method	Extracted DNA (ng/g)	DNA Quality		qPCR: Ct-Values		
		λ_260/280_	λ_260/230_	1:1	1:10	1:100
MP Soil	1935	1.43	0.02	n.d.	n.d.	38.61 *
MP Soil EtOH	1050	1.56	0.04	n.d.	34.23 *	38.10
AJ Soil 1	1184	0.97	0.02	n.d.	37.12 *	39.60
AJ Soil 2	448	0.72	0.01	n.d.	38.12	37.72 *
PeQLab Soil	1120	1.52	0.01	39.20	n.d.	37.82 *
QIA Mini slu	6560	1.08	0.29	n.d.	n.d.	n.d.
QIA Mini sup	1560	1.69	0.41	n.d.	n.d.	n.d.
QIA stool 1	19600	2.81	0.12	n.d.	n.d.	n.d.
QIA stool 2	19200	2.56	0.11	n.d.	n.d.	38.69 *
ZR Soil 1	7520	1.08	0.27	n.d.	39.73 *	n.d.
ZR Soil 2	13960	1.10	0.39	39.48	38.43 *	n.d.
Skim Milk	140	1.59	0.20	n.d.	39.09 *	39.58
PhChl	100	1.26	0.22	39.72 *	n.d.	n.d.
PhChl Skim 1	130	0.89	2.07	36.85 *	38.23	n.d.
PhChl Skim 2	170	1.00	0.49	35.57 *	37.00	n.d.
EURx Soil 0 Pol	1560	1.20	0.40	n.d.	n.d.	n.d.
EURx Soil 20 Pol	1410	1.51	0.41	n.d.	39.36 *	39.96
EURx Soil 40 Pol	1200	2.49	0.43	n.d.	n.d.	n.d.
EURx Soil 60 Pol	1410	0.82	0.35	n.d.	38.42 *	39.64

* Samples that were used in DGGE analysis; n.d., not detectable.

It was possible to visualize at least two and up to nine distinct bands in all lanes. It is very common to use a GC clamp in DGGE for sharp discrimination of bands that occur close together. The DGGE conditions such as the run time ratio and the urea gradient were optimized in previous experiments with the aim of separating the bands using the whole available length of the gel (Figure 1). The number of distinctive bands with and without the GC clamp was similar, but a nested PCR of the qPCR product led to a loss of bands (data not shown). Thus, we decided to use the qPCR products without a GC clamp directly for DGGE. A sufficient discrimination of bands was confirmed by sequencing, in which the DNA of all bands was successfully determined as single species DNA. Due to the low number of bands, the fact that all methods were performed as parallel approaches from the same aliquoted samples, and from the experience of former experiments (data not shown), all bands occurring on a similar level were considered as one species. 

It was shown that an additional cleaning procedure with ethanol (Figure 1, lane 2) may lead to a decreased number of detectable species. Parallel approaches as well as samples extracted with the same method but with different DNA concentrations also lead to different bar patterns (Figure 1, lanes 3, 4, 7, 8). After sequence analysis some of the obtained sequences could be assigned to the same species. Those different bar patterns may be caused by variations of the 16S rRNA gene within one species [18].

Despite of the fact that the samples were homogenized prior to aliquotation, there might be some differences regarding detectable bacterial species. Cell aggregations might occur. Especially those aggregations within biofilms are difficult to destroy during common homogenization. Lunsdorf *et al.* [19] described the phenomenon that microorganisms are able to create clay hutches that may inhibit extraction.

Only 1% of all occurring species in a sample are detectable with PCR-DGGE according to Muyzer *et al.* [20]. The limitation of the DGGE is determined by primer specificity and the fact that species that occur in low abundance can hardly be detected. The bands in DGGE might occur as weak, as it is not possible to see them. This results mainly from the logarithmic amplification of molecules in PCR. A hypothetical initial number of 100 DNA copies in sample A and 1000 copies in sample B (Δ900) results after 30 cycles of ideal PCR amplification with a hypothetical efficiency of 100% theoretically in 1E11 and 1E12 (Δ9E11) molecules, respectively. Since this difference is increasing after each cycle, the effect might influence the DGGE results of experiments with many PCR cycles. Due to the PCR-inhibition of coextracted molecules, the qPCR of this study became positive after 34 to 40 cycles. Even using modern methods such as 16S rRNA-based analysis in next generation sequencing, a PCR has to be performed. 

As negative control, a sample of water was treated and analyzed in the same way as all other samples. In the phenol-chloroform extraction experiment, skim milk powder was added to the negative control. The DNA of *Thermus thermophilus*, *Pseudomonas saccharophila*, and *Mitsuaria chitosanitabida* was extracted from the skim milk powder. It is well known that skim milk may contain different microbial species [21,22]. If those species are known it is possible to ignore these taxa within the evaluation of appropriate methods.

Altogether, 13 species were identified by sequence analysis. This low number of species is caused by the clay properties and is in agreement with already published results [23]. The majority of clay minerals are much smaller than bacteria and there is only little space between those minerals. This space is often filled by even smaller particles that also occur in the examined matrix [12].

The assignment of taxa to the method that was used to extract the DNA as well as the indication of minimal and maximal detectable species is outlined in Table 3. If two different bands obtained by DGGE were determined as the same species but with a different homology of the 16S rRNA gene according to the National Center for Biotechnology Information (NCBI) database entries, this taxon was considered as one species regarding the minimal number of detectable taxa and as two species regarding the maximal number.

A few species of gram-positive genera such as *Clostridium*, *Streptococcus*, or *Staphylococcus* could only be detected by single methods in which all of those methods contain a mechanical cell disruption step. This observation conforms to the expectation that it is much more challenging to extract DNA from gram-positive bacteria. The only gram-positive species that was identified after chemical lysis without any mechanical treatment was *Propionibacterium acnes*.

It was possible to identify up to nine bacterial species with the MP Soil kit and the AJ Soil kit as well as seven different taxa with the PeQLab Soil kit. Furthermore, with the AJ Soil kit and the PeQLab Soil kit at least two and three gram-positive species were extracted, respectively. There were only a few species that were detectable by use of almost all DNA extraction methods. This led to the conclusion that a parallel DNA extraction with several methods leads to an increased number of detectable species. Out of this study a combination of the MP Soil kit + PeQLab Soil kit (10 of 14 species) or AJ Soil kit + PeQLab Soil kit (9 of 14 species) is recommended to detect as many taxa as possible.

The methods that resulted in the highest amounts of extracted DNA are not those that could be recommended for population analysis in clays. In a few cases it was not even possible to amplify the DNA. That means that the DNA concentration is not a sufficient parameter to evaluate a DNA extraction method for population analysis.

**Table 3:** Detected species dependent on the applied method.

Species		MP Soil	MP Soil EtOH	AJ Soil 1	AJ Soil 2	PeQLab Soil	QIA Mini 1	ZR Soil 1	ZR Soil 2	Skim Milk	PhChl	PhChl Skim 1	PhChl Skim 2	EURx Soil 20 Pol	EURx Soil 60 Pol
*Sphingomonas melonis* ^1^		X	X	X	X	X									X
*Mitsuaria chitosanitabida* ^1^		X	X	X	X		X	X	X	X	X	X	X	X	
*Pseudomonas saccharophila*		X		X			X			X					
*Propionibacterium acnes*		X	X	X	X		X	X	X		X	X			
*Caulobacter leidyi* ^1^		X		X					X						
*Pelomonas aquatic* ^2^					X			X							
*Streptococcus pneumonia* ^1^					X	X									
*Bradyrhizobium elkanii* ^1^						X									X
*Staphylococcus epidermidis* ^1^						X									
*Streptococcus mitis*						X						X			
*Aquabacterium commune* ^1^						X			X		X		X		
*Thermus thermophiles* ^2^										X		X	X		
*Comamonas denitrificans*														X	
*Clostridium thiosulfatireducens* ^1^															X
different spezies	minimal	5	3	5	5	6	3	3	4	3	3	2 *	2 *	2	3
	maximal	9	6	9	7	7	4	4	6	4	3	5	4	2	3

^1^ ≤96% sequence homology; ^2^ 100% sequence homology; * species caused by skim milk powder were not considered.

There were two to five taxa identified by use of phenol-chloroform extraction. Regarding these results and the environmental impact of the unhealthy chemicals phenol and chloroform [24,25], the method can only be recommended as an additional procedure in case of the necessity to combine several methods. The use of Poly A as an additional reagent did not lead to the identification of an increased number of different taxa in this study. The surface of clay minerals is between 5 m^2^/g and 500 m^2^/g [9]. To saturate all charges of these particles, a very high amount of Poly A might need to be added.

## 4. Conclusions

In this study it was demonstrated for the first time that only a combination of several DNA extraction methods leads to the detection of a high number of bacterial species in matrices with natural nanoparticles. The amount of extracted DNA does not correlate with the number of detectable species and should not be applied as the only quality parameter for the evaluation of DNA extraction methods for population analysis. It is recommended to use several dilutions of extracted DNA for PCR if investigating challenging matrices such as clays. A methodology to avoid DNA capturing caused by charged surfaces and edges of clayey nanoparticles that could be tested in the future is a cation exchange [26] with monovalent ions that are not able to act as cation bridges.
